# Reimbursement of Lung Cancer in Turkey: A 10‐Year Single Center Study

**DOI:** 10.1002/cam4.71014

**Published:** 2025-07-11

**Authors:** Kerem Ensarioğlu, Berna Akıncı Özyürek, Metin Dinçer, Hızır Alı Gümüşler

**Affiliations:** ^1^ Department of Pulmonary Medicine, Faculty of Health Sciences Ankara Ataturk Sanatoryum Training and Research Hospital Ankara Turkey; ^2^ Department of Health Management, Faculty of Health Sciences Ankara Yıldırım Beyazıt University Ankara Turkey; ^3^ Department of Invoice Ankara Ataturk Sanatoryum Training and Research Hospital Ankara Turkey

**Keywords:** carcinoma, health care costs, health expenditures, lung

## Abstract

**Objective and Background:**

Lung cancer is the most common carcinoma reported worldwide. The burden of lung carcinoma on healthcare has been reported in many countries, while a real‐life report within Turkey has yet to be published. This study aims to present the available results from a tertiary center specializing in pulmonology.

**Methods:**

The study's population consisted of 199.112 patient admissions, which were reimbursed under the national healthcare system. After exclusion criteria, 4.991 patients were evaluated in the study. The patient's demographic data, malignancy types, diagnostic methods for lung carcinoma type and staging, and treatment modalities were recorded.

**Results:**

The average healthcare expenditure during the treatment duration was 4289.4 (± 3739.4) USD, and the median was 3219.9 USD. The average expenditure was higher for male patients (3260 to 2977 USD, *p* = 0.003). Survivors had an overall lower healthcare expenditure than the non‐survivor group (2865 to 4230 USD). Patients with locally advanced disease classification also had a higher expenditure on other stages (*p* < 0.001). This was not present in SCLC, as those diagnosed with SCLC had the most expenditure at the limited stage (*p* < 0.001). Carcinoid tumors had the least expenditure compared to other subtypes at limited and advanced stages (*p* < 0.001). SCLC had the highest expenditure requirement at the limited stage (*p* < 0.001). At the locally advanced stage, large cell carcinoma, SCC, and adenocarcinoma were the subtypes requiring the highest expenditure (*p* < 0.001).

**Conclusion:**

The parameters affecting overall cost were age, gender, stage, and malignancy subtype. The healthcare expenditure was also affected by the difficulty of diagnosis, with the non‐diagnostic patient group having an overall higher cost. A longer duration of hospitalization and treatment given on an inpatient basis also contributed to a higher cost of care.

## 1 Introduction

Lung cancer is the most common carcinoma reported worldwide, according to the World Health Organization's cancer statistics. In 2023, an estimated 2.5 million new LC cases occurred worldwide, representing 12.4% (1.4 million) of all new cancer diagnoses [1]. In addition to being the most diagnosed cancer type, it is also the most common cause of cancer death, a statement of its overall mortality and morbidity [1]. In Turkey as well, lung cancer remains the most commonly observed cancer type, with the highest overall mortality [2]. Regarding the Turkish Statistics Institute (TSI) report, the combined mortality of “larynx and trachea/bronchus/lung neoplasm” was 29.7% in 2021 and 29.4% in 2022, when evaluated as a proportion of deaths from malignant neoplasms. As for their distribution in overall mortality, malignancy within these years was responsible for 14% and 15.2% of all causes of death, respectively [3]. As for subtype distribution, 85% of lung carcinoma is defined as non‐small lung carcinoma (NSCLC), while the remaining 15% is categorized as small cell lung carcinoma (SCLC), which has higher mortality and morbidity compared to the NSCLC group [4].

As seen both worldwide and within Turkiye, the global lung cancer burden continues to increase owing to population growth, population aging, and changes in the prevalence of risk factors (e.g., smoking and air pollution), which will further exert tremendous strain on populations and health systems worldwide [5]. This trend appears to be changing, with possible new treatment modalities, as in the United States of America (USA), the overall trend has begun decreasing [6].

Overall, lung malignancies lead to a higher socioeconomic burden, as seen in the United States of America (USA) and European Union (EU) reports, in which a recent study in the USA stated a loss of 21.3 billion USA dollars regarding workforce loss out of a total 94.4 billion dollar loss attributed to all cancer‐related losses [7]. Similarly, in the EU, lung carcinoma is the leading healthcare expenditure requirement; out of the 126 billion euros budget for all carcinomas, 18.8 billion euros (15% of the budget) was spent on lung carcinoma.

Currently, no definitive data are available for lung carcinoma and carcinoma‐related expenditure. The study aims to investigate the overall spending for bronchial and lung carcinoma.

## 2 Methods

The study's population consisted of patients evaluated at a tertiary hospital specializing in pulmonary medicine between 2013 and 2022. A record of 199,112 patient admissions whose was under national healthcare insurance was evaluated for the study.

**FIGURE 1:**
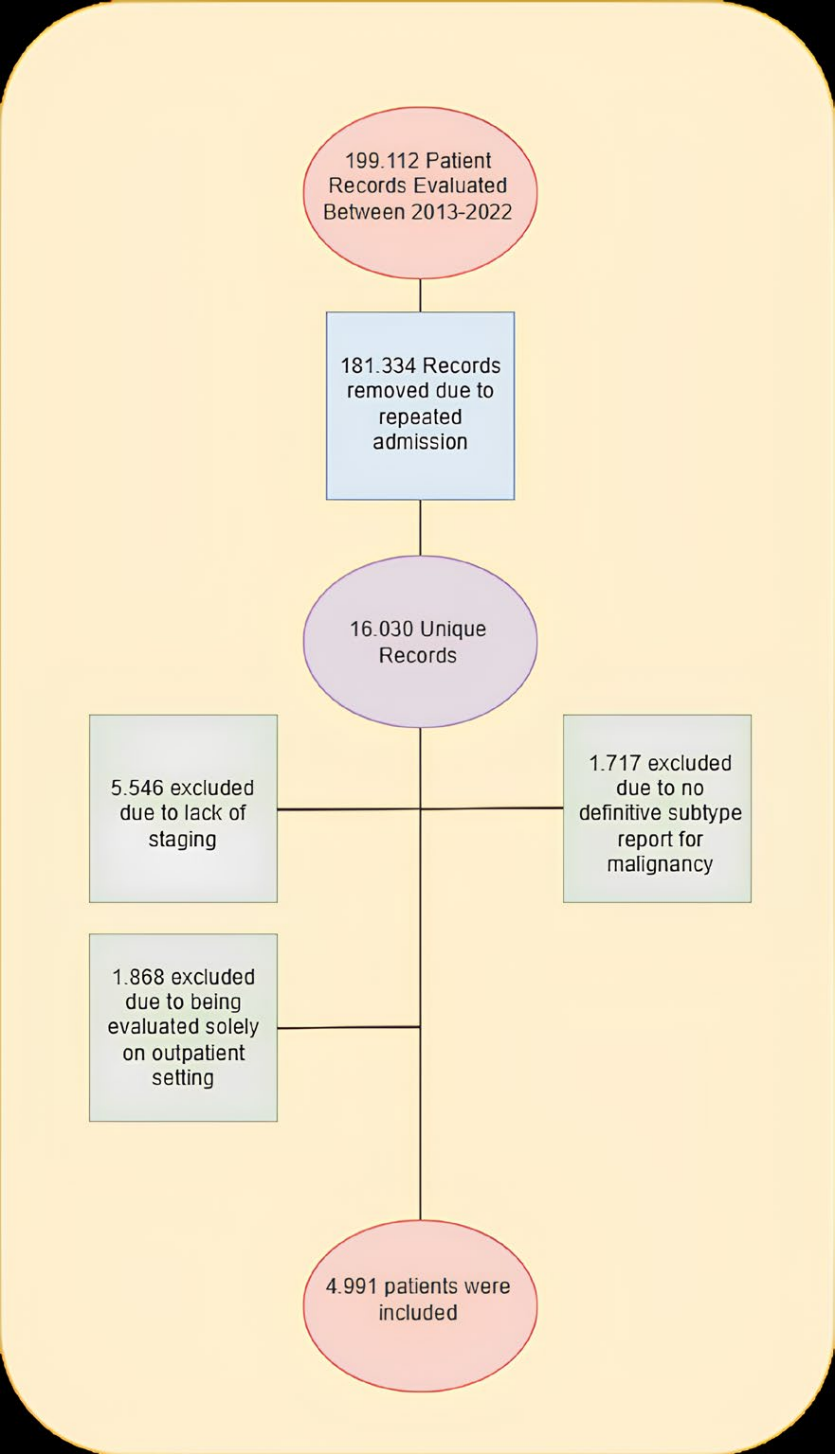
Patient selection flow chart for the study. After exclusion of repeated records and removal of patients with lacking stage and subtype information, the remaining 4991 patients were included in the study.

After the removal of 181,334 patient admissions from the database due to repeated admissions, additional yet irrelevant system data entries, and other supportive data queries, 16,030 unique patients were found. According to the exclusion criteria, a total of 11,039 patients were excluded. 5.546 patients were excluded due to lack of stage and malignancy type, 2008 patients were excluded due to lack of staging, and 1717 patients were removed as no definitive subtype report for malignancy could be reached. Exclusion of 1868 patients was made due to the patients being evaluated solely on an outpatient basis. The remaining 4991 patients were included in the study (Figure 1). Confirmation of the data and distribution of the subgroups was performed by two authors, one responsible for the billing section and had access to data that was utilized for expenditure, while the other author, a medical doctor specialized in pulmonary medicine, had access to hospital records, including the national database. In cases where two system records did not match, such as in the case of a patient undergoing chemotherapy yet not having a diagnosis on either record, the national database records were accessed for validation.

The patients' demographic data, malignancy types, diagnostic methods utilized for lung carcinoma type and staging (which includes surgical, transthoracic needle aspiration, and endobronchial ultrasonography), treatment modalities used (chemotherapy, radiotherapy, palliative care, and antibiotic regimens) were recorded. The expenditure for these interventions and their counts were additionally recorded. The lung carcinoma staging was divided into local, locally advanced, advanced, and metastatic.

Survival in lung carcinoma is mainly dependent on the initial staging; as such, proper staging methods constitute the cornerstone of lung carcinoma management. The stage of a patient, combined with the type of malignancy, determines available treatment modalities. Currently, the initial staging approach would be to investigate the involvement of mediastinal structures and assess if extrathoracic tumor presence exists, in which a combination of medical history, symptoms, imaging modalities, minimally invasive approaches, and surgical intervention is utilized.

This was followed up by an invasive method of sampling, depending on the imaging results and status of the patient, as severe or repeated methods of sampling for a patient who is not a candidate for chemotherapy or surgical resection would be detrimental to the overall status of the patient. The mentioned factors, including stage, a subtype of malignancy, diagnostic methods utilized, and hospitalization requirements, were all accepted as factors contributing to the overall cost of health expenditure.

The largest purchaser of health services in Turkey is the Social Security Institution. While determining the costs, fees for service were taken into account based on the Health Implementation Communiqué Prices issued by the Social Security Institution, which determines the tariff of health services. All expenditure data was received as Turkish Lira (TL) and then changed to United States Dollar (USD) using the reference of the yearly exchange rate provided by the Central Bank of the Republic of Turkey's archive [8].

Overall, patients' diagnosis, demographic information, localization of treatment, diagnosis subtype, stage at diagnosis, and survival were expected as parameters that might affect overall expenditure and were included in the statistical evaluation.

The study was performed after the approval of the hospital ethics committee, which acts as the Institutional Review Board (IRB), with additional ethics approval given by the Faculty of Health Sciences for the utilization of healthcare expenditure data (Approval date 03.01.2023, approval number 23‐1315). The ethics committee granted an exemption from the written consent of the patients due to the data evaluation being retrospective in nature.

The patients' results were put into a Microsoft Excel file for overall evaluation. After investigating any mis‐input and values, the data were moved to a statistics module (IBM SPSS Version 25th for Windows). The initial assessment was performed by descriptive analysis, for which values were given with mean and standard deviation or with median and percentiles as required. Parametric distribution was evaluated using a Q‐Q plot analysis. Mann–Whitney *U* and chi‐square tests were utilized to evaluate the difference between groups. *p* values at or below 0.05 were accepted as statistically significant.

## 3 Results

A total of 4991 patients with lung malignancy were included in the study, with 582 (11.7%) patients female and the remaining 4409 (88.3%) male patients. The average age was 70.2 (± 9.9), with a range between 25 and 101 years. Most patients (*n* = 3485, 69.8%) had solely inpatient treatment history, while the remaining (*n* = 1506, 30.2%) had inpatient and outpatient treatment history.

Non‐small cell lung carcinoma (NSCLC) was the most observed subtype, with squamous cell carcinoma (SCC) being the most common reported type (*n* = 1841, 36.9%), followed by adenocarcinoma (*n* = 1455, 29.2%). Small cell lung carcinoma (SCLC) was seen as the third most observed malignancy (*n* = 1041, 20.9%), while carcinoid tumors were the rarest (*n* = 19, 0.4%).

Less than 2.5% of patients (*n* = 114, 2.3%) had no definite diagnosis and were accepted as a non‐diagnostic group. This group included patients whose definitive diagnosis could not be reached, regardless of possible interventions performed, which at least required one interventional method in which an inadequate diagnosis was reached. Most of the patients had been either with an advanced stage (*n* = 3054, 61.2%) or metastatic (*n* = 912, 18.3%) at the time of diagnosis. Local advanced disease classification was found at 7.3% (*n* = 364), while 13.2% of the patients were classified as limited stage. As of the study's completion, 3541 (70.9%) patients were alive, while 1450 (29.1%) patients were lost (Page 10).

When evaluated for age, patients who required solely inpatient treatment were younger than those requiring inpatient and outpatient care (69 to 70, *Z* = 9.623; *p* < 0.001). Similarly, patients with limited stage were younger than other groups, and patients with local advanced stage were younger than those accepted as advanced or metastatic stage (*χ* ^2^ = 123.467; *p* < 0.001). Living patients were older than those lost; this was statistically significant. However, the median age difference was limited (70 to 69.5, *Z* = 2.216; *p* = 0.027). Carcinoid tumor was observed at a younger age compared to NSCLC, SCC, and malignant epithelial tumors, while large cell carcinoma patients were also younger than those with SCC and malignant epithelial tumors (*χ* ^2^ = 44.989; *p* < 0.001) (Table 1).

**TABLE 1:** Patient demographic, treatment localization, malignancy subtype, and stage distribution according to age.

Parameters	*n* (4991)	%	Median age (IQR)	Comparison
Gender				
Female	582	11.7	69.0 (15.0)	*Z* = 1.294; *p* = 0.196
Male	4409	88.3	70.0 (13.0)	
Treatment location				
Inpatient and outpatient	1506	30.2	73.0 (13.0)^b^	*Z* = 9.623; *p* < 0.001
Inpatient	3485	69.8	69.0 (13.0)^a^	
Malignancy subtype				
Adenocarcinoma	1455	29.2	69.0 (13.0)^e^	*χ* ^2^ = 44.989; *p* < 0.001
Carcinoid tumor	19	0.4	58.0 (15.0)^a^	
Large cell carcinoma	40	0.8	65.5 (9.0)^c^	
Malign epithelioma	211	4.2	72.0 (16.0)^b,d^	
Non‐diagnostic	114	2.3	71.0 (11.0)^b^	
Non‐small cell carcinoma	268	5.4	70.0 (13.0)^b^	
Squamous cell carcinoma	1843	36.9	71.0 (14.0)^b,d,f^	
Small cell carcinoma	1041	20.9	70.0 (14.0)	
Stage				
Limited	661	13.2	67.0 (12.0)^a^	*χ* ^2^ = 123.467; *p* < 0.001
Local advanced	364	7.3	70.0 (13.0)^b,c^	
Advanced	3054	61.2	71.0 (14.0)^b,d^	
Metastatic	912	18.3	70.0 (13.0)^b,d^	
Survival				
Survivor	3541	70.9	70.0 (13.0)^b^	*Z* = 2.216; *p* = 0.027
Non‐survivor	1450	29.1	69.5 (13.0)^a^	

*Note:* Different groups were reported as: a < b, c < d, e < f.

Abbreviations: *Z*, Mann–Whitney test; *χ* ^2^, Chi‐square.

The hospitalization duration varied from 1 day to 236, with a mean duration of 33 (± 24.1) days and a median of 26 days. Male patients had a longer hospitalization duration compared to female patients (23 to 27 days, *Z* = 5.032; *p* < 0.001), and those with solely inpatient hospitalization history had a longer total hospitalization duration compared to those receiving both inpatient and outpatient care (69.8 to 30.2 days, *Z* = 16.667; *p* < 0.001). The non‐diagnostic patient group had a longer hospitalization duration than all other defined subtypes (*χ* ^2^ = 53.265; *p* < 0.001). Patients at a locally advanced stage also required a longer overall hospitalization duration than the other stages (*χ* ^2^ = 267.441; *p* < 0.001). Survivors had a shorter hospitalization history (23 to 36 days, *Z* = 19.443; *p* < 0.001) (Table 2).

**TABLE 2:** Patient demographic, treatment localization, malignancy subtype, and stage distribution according to admission days.

Parameters	*n* (4991)	%	Admission days	Comparison
			Median (IQR)	
Gender				
Female	582	11.7	23.0 (24.0)^a^	*Z* = 5.032; *p* < 0.001
Male	4409	88.3	27.0 (26.0)^b^	
Treatment location				
Inpatient and outpatient	1506	30.2	22.0 (17.0)^a^	*Z* = 16.667; *p* < 0.001
Inpatient	3485	69.8	30.0 (30.0)^b^	
Malignancy subtype				
Adenocarcinoma	1455	29.2	25.0 (25.0)^c^	*χ* ^2^ = 53.265; *p* < 0.001
Carcinoid tumor	19	0.4	15.0 (22.0)^e^	
Large cell carcinoma	40	0.8	22.0 (16.8)	
Malign epithelioma	211	4.2	24.0 (22.0)^a^	
Non‐diagnostic	114	2.3	33.5 (35.3)^b,d,f^	
Non‐small cell carcinoma	268	5.4	27.0 (23.8)	
Squamous cell carcinoma	1843	36.9	27.0 (28.0)^b^	
Small cell carcinoma	1041	20.9	28.0 (24.0)^b,d^	
Stage				
Limited	661	13.2	21.0 (24.0)^a^	*χ* ^2^ = 267.441; *p* < 0.001
Local advanced	364	7.3	42.0 (38.8)^b,d,f^	
Advanced	3054	61.2	25.0 (23.0)^b,c^	
Metastatic	912	18.3	34.0 (30.0)^b,d,e^	
Survival				
Survivor	3541	70.9	23.0 (23.0)^a^	*Z* = 19.443; *p* < 0.001
Non‐survivor	1450	29.1	36.0 (31.0)^b^	

*Note:* Different groups were reported as: a < b, c < d, e < f.

Abbreviations: *Z*, Mann–Whitney test; *χ* ^2^, Chi‐square.

The average healthcare expenditure for a patient diagnosed with lung malignancy during the treatment duration was 4289.4 (± 3739.4) USD, and the median was 3219.9 USD. The average expenditure was higher for male patients (3260 to 2977 USD, *Z* = 2.957; *p* = 0.003). The total expenditure for patients requiring solely inpatient care was nearly double that of patients receiving inpatient and outpatient care. Survivors had an overall lower healthcare expenditure than the non‐survivor group (2865 to 4230 USD). Similar to the hospitalization duration comparison, the non‐diagnostic patient group had a higher healthcare expenditure than nearly all other subtypes (*χ* ^2^ = 66.600; *p* < 0.001). Patients with a locally advanced disease classification also had a higher expenditure than other stages (*χ* ^2^ = 665.662; *p* < 0.001) (Table 3).

**TABLE 3:** Healthcare expenditure according to demographic, treatment localization, malignancy subtype, and stage distribution.

Parameters	*n* (4991)	%	Expenditure (USD)	Comparison
			Median (IQR)	
Gender				
Female	582	11.7	2977.1 (3741.0)^a^	*Z* = 2.957; *p* = 0.003
Male	4409	88.3	3260.5 (3870.3)^b^	
Treatment location				
Inpatient and outpatient	1506	30.2	4080.6 (4177.0)^b^	*Z* = 27.668; *p* < 0.001
Inpatient	3485	69.8	1913.0 (1803.6)^a^	
Malignancy subtype				
Adenocarcinoma	1455	29.2	3192.1 (3704.1)^b,k^	*Z* = 66.600; *p* < 0.001
Carcinoid tumor	19	0.4	2080.4 (1916.5)^c^	
Large cell carcinoma	40	0.8	3737.6 (4021.1)^b^	
Malign epithelioma	211	4.2	2469.8 (2920.1)^a^	
Non‐diagnostic	114	2.3	4597.3 (3887.1)^b,d,h,j,l,n^	
Non‐small cell carcinoma	268	5.4	2839.1 (3201.6)^e,g,I^	
Squamous cell carcinoma	1843	36.9	3403.0 (4425.9)^b,f^	
Small cell carcinoma	1041	20.9	3140.5 (3444.3)^b,m^	
Stage				
Limited	661	13.2	4455.7 (4529.5)^b,d,e^	*χ* ^2^ = 665.662; *p* < 0.001
Local advanced	364	7.3	5799.2 (4521.7)^b,d,f^	
Advanced	3054	61.2	2531.1 (3016.7)^a^	
Metastatic	912	18.3	3945.1 (4059.8)^b,c^	
Survival				
Survivor	3541	70.9	2865.5 (3487.9)^a^	*Z* = 14.468; *p* < 0.001
Non‐survivor	1450	29.1	4230.8 (4421.8)^b^	

*Note:* Different groups were reported as: a < b, c < d, e < f, g < h, i < j, k < l, m < n.

Abbreviations: *Z*, Mann–Whitney test; *χ* ^2^, Chi‐square.

The observation of locally advanced disease being the highest expenditure group was present among all subtypes; however, it was more prominent in SCC and adenocarcinoma subtypes (*χ* ^2^ = 311.197; *p* < 0.001 and *χ* ^2^ = 311.197; *p* < 0.001, respectively). This was not present in SCLC, as those diagnosed with SCLC had the most expenditure at the limited stage (*χ* ^2^ = 95.778; *p* < 0.001) (Table 4).

**TABLE 4:** Healthcare expenditure according to malignancy subtype and relevant stage.

Malignancy type and stage	*n* (4991)	Expenditure (USD)	Comparison
		Median (IQR)	
Adenocarcinoma	1455		
Limited	244	3994.1 (3676.0)^b,e^	*χ* ^2^ = 194.904; *p* < 0.001
Locally advanced	92	6325.9 (5065.4)^b,d,f^	
Advanced	771	2427.4 (2813.7)^a^	
Metastatic	348	3942.2 (3984.6)^b,c^	
Carcinoid tumor	19		
Limited	8	2260.7 (1214.4)	*Z* = 1.636; *p* = 0.114
Locally advanced	1	N/A	
Advanced	9	1163.0 (2128.6)	
Metastatic	1	N/A	
Large cell carcinoma	40		
Limited	6	7113.3 (6467.6)	*Z* = 1.596; *p* = 0.117
Locally advanced	0	N/A	
Advanced	33	3693.8 (3539.4)	
Metastatic	1	N/A	
Malign epithelioma	211		
Limited	1	N/A	*χ* ^2^ = 16.038; *p* = 0.001
Locally advanced	11	4438.9 (2722.1)^b^	
Advanced	161	1960.9 (2683.0)^a^	
Metastatic	38	3068.5 (2984.7)	
Non‐diagnostic	114		
Limited	1	N/A	*Z* = 1.286; *p* = 0.198
Locally advanced	27	4910.7 (4406.3)	
Advanced	0	N/A	
Metastatic	86	4427.3 (4161.7)	
Non‐small cell carcinoma	268		
Limited	0	N/A	*χ* ^2^ = 35.599; *p* < 0.001
Locally advanced	24	5811.4 (4450.4)^b,d^	
Advanced	164	2237.9 (2788.8)^a^	
Metastatic	80	3200.3 (2849.7)^b,c^	
Squamous cell carcinoma	1843		
Limited	303	4736.5 (5119.0)^b,c^	*χ* ^2^ = 311.197; *p* < 0.001
Locally advanced	201	6045.4 (4378.3)^b,d^	
Advanced	1043	2462.6 (3220.6)^a^	
Metastatic	296	4187.1 (4904.5)^b,c^	
Small cell carcinoma	1041		
Limited	98	5796.6 (4188.3)^b,d^	*χ* ^2^ = 95.778; *p* < 0.001
Locally advanced	8	5068.9 (4426.7)	
Advanced	873	2862.9 (3082.9)^a^	
Metastatic	62	4008.3 (3055.6)^b,c^	

*Note:* Different groups were reported as: a < b, c < d, e < f.

Abbreviations: *Z*, Mann–Whitney test; *χ* ^2^, chi‐square.

When malignancy subtypes were evaluated at the same stages in terms of expenditure, carcinoid tumors had the least expenditure compared to other subtypes at limited and advanced stages (*χ* ^2^ = 42.060; *p* < 0.001). SCLC had the highest expenditure requirement at the limited stage (*χ* ^2^ = 34.257; *p* < 0.001). Adenocarcinoma had the highest expenditure at the locally advanced stage; however, the observation was not statistically significant (*χ* ^2^ = 7.905; *p* = 0.245).

At the locally advanced stage, large cell carcinoma, SCC, and adenocarcinoma were the subtypes requiring the highest expenditure (*χ* ^2^ = 42.060; *p* < 0.001). In metastatic patients, a statistically significant difference was present between only SCC and NSCLC patients, with SCC patients requiring less expenditure (*χ* ^2^ = 21.557; *p* = 0.003). Despite not being statistically significant, the highest expenditure need was for the non‐diagnostic group, while the lowest requirement was for patients diagnosed with malignant epithelial tumors (Table 5). A very weak negative correlation was present between age and total healthcare expenditure (Rho = −0.155; *p* < 0.001). A strong positive correlation was present between total admission days and healthcare expenditure (Rho = 0.660; *p* < 0.001).

**TABLE 5:** Healthcare expenditure according to stage and malignancy subtype.

Malignancy type and stage	*n* (4991)	Expenditure (USD)	Comparison
		Median (IQR)	
Limited	661		
Adenocarcinoma	244	3994.1 (3676.0)^c^	*χ* ^2^ = 34.257; *p* < 0.001
Carcinoid tumor	8	2260.7 (1214.4)^a^	
Large cell carcinoma	6	7113.3 (6467.6)	
Malign epithelioma	1	N/A	
Non‐diagnostic	1	N/A	
Non‐small cell carcinoma	0	N/A	
Squamous cell carcinoma	303	4736.5 (5119.0)^b^	
Small cell carcinoma	98	5796.6 (4188.3)^b,d^	
Local advanced	364		
Adenocarcinoma	92	6325.9 (5065.4)	*χ* ^2^ = 7.905; *p* = 0.245
Carcinoid tumor	1	N/A	
Large cell carcinoma	0	N/A	
Malign epithelioma	11	4438.9 (2722.1)	
Non‐diagnostic	27	4910.7 (4406.3)	
Non‐small cell carcinoma	24	6045.4 (4378.3)	
Squamous cell carcinoma	201	5068.9 (4426.7)	
Small cell carcinoma	8	5811.4 (4450.4)	
Advanced	3054		
Adenocarcinoma	771	2427.4 (2813.7)^j,k^	*χ* ^2^ = 42.060; *p* < 0.001
Carcinoid tumor	9	1163.0 (2128.6)^a^	
Large cell carcinoma	33	3693.8 (3539.4)^b,d,f,l,q^	
Malign epithelioma	161	1960.9 (2683.0)^c^	
Non‐diagnostic	0	N/A	
Non‐small cell carcinoma	164	2462.6 (3220.6)^e,h^	
Squamous cell carcinoma	1043	2862.9 (3082.9)^n,o^	
Small cell carcinoma	873	2237.9 (2788.8)^d,g,I,m^	
Metastatic	912		
Adenocarcinoma	348	3942.2 (3984.6)	*χ* ^2^ = 21.557; *p* = 0.003
Carcinoid tumor	1	N/A	
Large cell carcinoma	1	N/A	
Malign epithelioma	38	3068.5 (2984.7)	
Non‐diagnostic	86	4427.3 (4161.7)	
Non‐small cell carcinoma	80	4187.1 (4904.5)^b^	
Squamous cell carcinoma	296	4008.3 (3055.6)^a^	
Small cell carcinoma	62	3200.3 (2849.7)	

*Note:* Different groups were reported as: a < b, c < d, e < f, g < h, i < j, k < l, m < n, o < q.

Abbreviations: *Z*, Mann–Whitney test; *χ* ^2^, chi‐square.

## 4 Discussion

The study aimed to investigate the economic burden of lung carcinoma by evaluating patients diagnosed with it within 10 years in one of the four most prominent pulmonology centers in our country. The socioeconomic burden of lung carcinoma varies from country to country, depending on the country's economic status, healthcare system, and purchasing power [9]. Average age, sociocultural status, industrial status, and geographic factors also contribute to the overall healthcare expenditure provided for malignancy care. The increase in the elderly population is also a factor, as it results in the overall increased healthcare services provided to them, which leads to more cases being diagnosed. Both these findings naturally contribute to a higher healthcare burden. The overall age of 70‐year‐old patients in our study also supports this fact. This observation was independent of gender, as the age did not vary within genders.

The majority of the patients who had received care solely in hospital admission was a factor that is attributed to the dominance of patients with higher carcinoma stage, which in many cases required a longer and more intensive treatment regimen along with control of more complications that may be observed during the period. This observation also supports the presumption that patients with a better functional status were able to be managed in an outpatient setting after an initial hospitalization period. It is of important note to state that at least one inpatient admission was required in our study, as stated in the exclusion criteria, due to the nature of the reimbursement system in Turkey, as for diagnostic procedures at least one inpatient day of admission is required, even in cases where a patient is later discharged within the same day.

The observance of NSCLC as the most prominent type reported in the study was a result that is accepted as the natural distribution of lung carcinoma. The distribution of subtypes was also within expected parameters, such as carcinoid tumors being reported as the rarest type. The same could also be stated for patients with no definitive diagnosis, as they were reported to be below 2.5%, which was within acceptable ranges for diagnostic processes. The distribution of stages was also correlated with available data on lung carcinoma, as most patients were either in the advanced stage or metastatic. These results strongly support the validity of our results when compared to available observations in the literature [10, 11].

The age difference between patients requiring solely inpatient treatment and those with both inpatient and outpatient treatment history was another point of note, as patients in the inpatient treatment group were younger than those requiring both approaches.

While this may warrant the previously mentioned assumption that those requiring hospitalization had a worse clinical outcome than those who could have been managed in an outpatient setting, the fact is that patients who were within the limited stage and those considered locally advanced stage were also younger than those in the metastatic state. Thus, it was also possible to consider that the elderly patients, at some point in care, were discharged for the care given in the home or were not considered for more invasive treatment modalities requiring hospitalization.

This was further supported by the observation that survivors had shorter hospitalization durations. The dominance of younger patients with higher hospitalizations, on the other hand, leads to a presumption that younger patients were better suited for surgery and required a more extended period of ward follow‐up afterward. The overall more extended hospitalization for the non‐diagnostic group was expected due to repeated diagnostic interventions performed on these patients. Similarly, locally advanced patients' longer stay was attributed to the radiotherapy requirement, as in most cases, patients had to be admitted during the radiotherapy period.

The longer duration of admittance for male patients and higher expenditure for the same gender were other significant factors indicating the role of gender in overall healthcare expenses. The observation of patients solely requiring inpatient care requiring nearly double the amount of expenditure to the group receiving inpatient and outpatient care supports the former statement that most of these patients had to be hospitalized for procedures requiring extra expenditure, such as more intervention instead of basic medical care.

For most diagnostic interventions, it was possible to perform them on an outpatient basis; however, as seen in the study, the preference was to admit the patients to the ward beforehand. Similarly, control of complications was another aspect that could have been performed without admission. The mean duration of hospitalization was around 1 month, which reflects both points, stating that admission was utilized not only for diagnostic methods but also for complication control and assessment.

An overall increase in admission duration for patients diagnosed with locally advanced disease also supports this assumption, as those patients were often treated with chemoradiotherapy and had more complications attributed to both advanced disease and more intensive regimens required. The malignancy subtype also affected the overall cost, with SCLC having a lower expenditure at the advanced stage compared to other subtypes, while having a higher expenditure at the limited stage. This was attributed to a more palliative approach and limited treatment modalities available for SCLC as the stage increases compared to other subtypes. Treatment modalities in the current literature have been a topic of discussion, with studies on possible resistance patterns of available immunotherapy among NSCLC and other possible factors that might affect resistance. These factors may not be so evident in the case of SCLC, as stated earlier, current available treatment options remain limited compared to NSCLC, and hence, a higher cost of care in the limited stage, while lower in the advanced stage [12, 13]. An additional factor that might affect overall cost would be the mentioned availability of immunotherapy and targeted treatments, and their contribution to the overall cost.

A study of the cost of care in China showed that healthcare expenditures were highest within the first year following diagnosis, and the highest expenditures were related to hospital admission costs. The average economic burden per patient was reported to be 43,336 US dollars [9]. Another study performed in South Korea stated that the cancer burden among healthcare expenditures was 17% of the total budget, accounting for 3.3 billion US dollars. Among expenditures for malignancy, 28.3% was attributed to healthcare personnel expenses, 17.2% was non‐medical bureaucratic related, 24.2% was spent for morbidity control, and the remaining 30.3% was related to end‐of‐life care and other mortality‐related spending [14]. A Canadian study of 24,729 patients reported an overall cost of 1.9 Canadian Dollars for the patient's care, with the most expenditures spent on hospital admission, followed by outpatient follow‐up visits and consultation fees [15]. The total per‐patient cost of care of advanced NSCLC in Spain was estimated to range from €11,301 to €32,754, depending on the number of treatments received [16]. These reported values were relatively lower than what the study stated regarding our parameters, with an average of 3219.9 USD, roughly a difference of 10 times.

A USA‐based study reported a mean expenditure of 35,141 US dollars for patients diagnosed with lung cancer [17]. Overall, an expenditure of 21.1 billion US dollars in 2015 and 23.8 billion US dollars in 2020 was spent on lung cancer management alone in the USA, as declared by the National Cancer Institute. For the Middle East, expenditure varied by the country, as stated in the study of Horfmacher et al., which included select countries in Africa as well [18]. While no direct cost was stated in this study, a yearly per capita cost was given, with the highest present in Kuwait (79 US dollars per capita) and the lowest in Egypt (11 dollars per capita).

This severe difference could be explained by a myriad of reasons: healthcare expenditure of malignancy being entirely supported by the state, differences in currencies, and the gross domestic product purchasing power varying between countries could be counted for [19].

The main limitations of the study could be summarized as patient selection and staging. While including an adequate number for statistical evaluation, patient selection excluded patients who had received care only on an outpatient basis. The main reasoning was that a patient had to be hospitalized in our hospital setting for reimbursement due to the relatively high risk of interventions performed on already fragile patients. As a single‐center study, this limitation also affected patient heterogeneity, with contributing factors that might have affected results, such as Turkey having a higher SCLC rate than the USA, which might affect the generalizability of the results. While the center remains one of the most prominent hospitals for lung carcinoma management, generalization of the study to the country might not be appropriate, especially in resource‐limited settings. Staging was also another issue, as absolute staging reports, such as 3A and 3B for respective malignancy subtypes, could not be performed due to the majority of former patient consultations and reports being unavailable. As such, a former staging could be performed by dividing stages into four main categories of limited, local advanced, advanced, and metastatic. This division was considered adequate for statistical and expenditure purposes, as it also fits the available literature. However, the unorthodox and non‐standard staging evaluation remains a limiting factor for the generalizability of the study.

Another limitation was the increase in cost trend over time, or rather, the estimation of inflation in the evaluation. To exclude the varying inflation present in the Turkish Lira during the mentioned time period, expenditures within their respective years were changed to USD, using the average exchange rate within the year. Due to this estimation method, the inclusion of inflation of USD could not be reliably included in the study. This raw‐USD‐based analysis could have contributed varying results when compared internationally. Survival evaluation was another factor that could not be detailed reported, as survival data were mainly received from the national healthcare base, in which information regarding follow‐up periods was not available. While such data was present for some patients who had received their care solely from our institution, for patients lost during admission at other centers or had been lost due to non‐pulmonary causes, an exact estimation of survival analysis could not be made. Similarly, while multiple group comparisons were performed, a multivariate analysis could not be performed to evaluate potential confounders as per the design of the study, with available parameters being mostly present with median values due to varying ranges of expenditure.

Regarding expenditure differences besides malignancy subtype, such as administrative costs for different subtypes or drug expenditure, an exact statement could not be made. This was attributed due to the study design, as while total cost of said expenditures were known, such as diagnostic modalities, subgrouping could not be performed, as many cases did not have corresponding subdivisions in the billing system to cross‐validate. As such, implantation of these to possible actional strategies may remain limited and require further specific studies.

**FIGURE 2:**
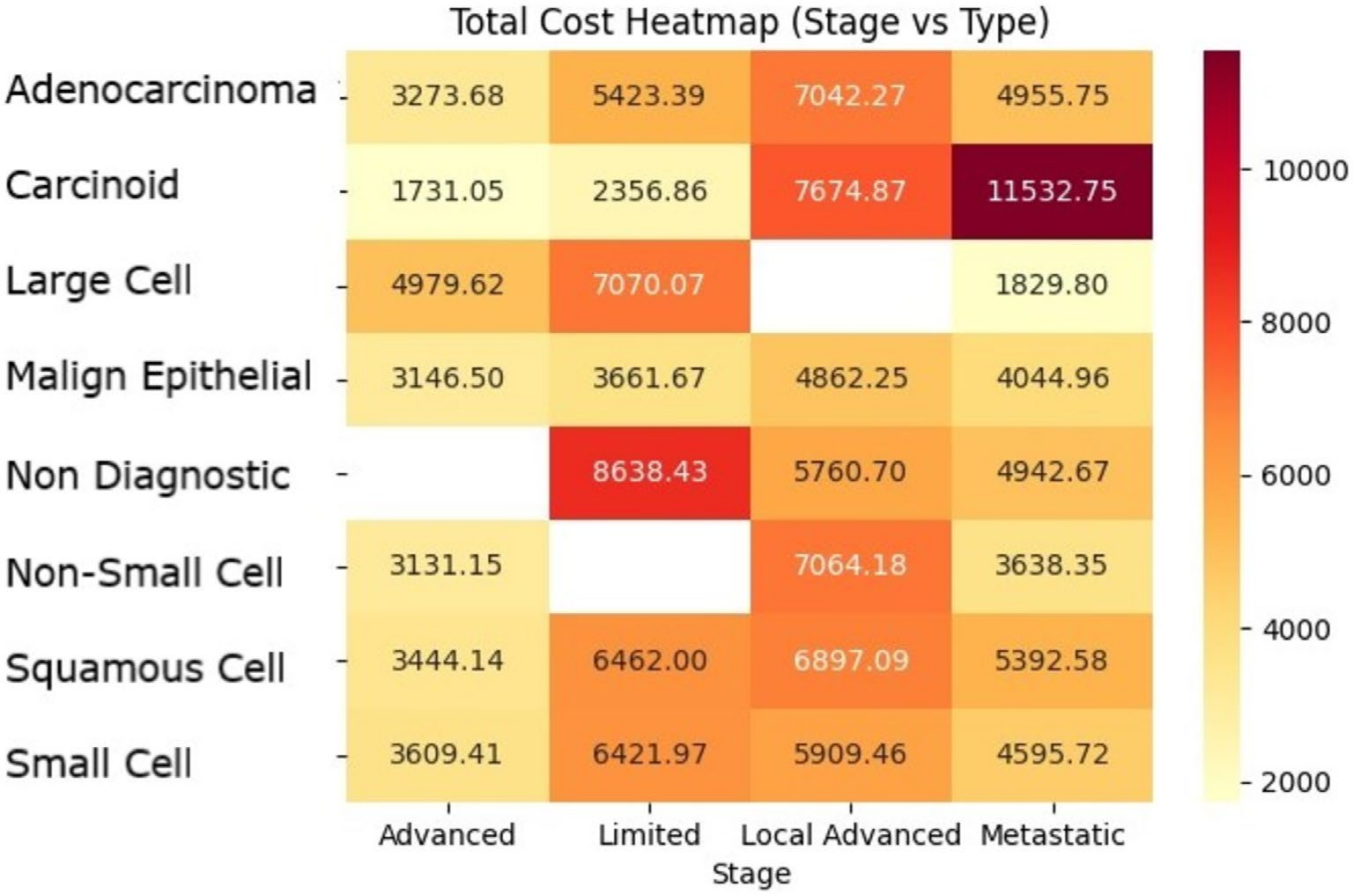
Heatmap of stage and subtype expenditure. In the heatmap analysis, the distribution of malignancy‐related healthcare expenditure is presented according to stage and subtype. The least expenditure was observed among the early stages of carcinoid tumor, while the non‐diagnostic group had the highest expenditure, even among the early stages of malignancy.

Regardless, the available data suggest that early disease control remains paramount for overall expenditure, which was more prominent in NSCLC. While not evident for SCLC compared to other subtypes, such as the massive leap in carcinoid tumor, we believe early diagnosis is essential for not only patient healthcare but also for economic benefit to the overall healthcare system (Figure 2).

## 5 Conclusion

Parameters affecting overall cost were found to be age, gender, stage, and malignancy subtype, with varying roles of malignancy present for different subtypes. Similarly, the role of the stage upon overall cost also varied depending on the malignancy subtype. The study also reflected the difficulty of diagnosis, with the non‐diagnostic patient group having an overall higher cost. A longer duration of hospitalization and treatment being given on an inpatient basis also contributed to a higher cost of care. The prevention of lung malignancy remains the most optimal method to reduce health expenditure, with the smoking cessation strategies being the most reliable and least expensive method.

## Author Contributions

**Kerem Ensarioğlu:** data curation (equal), formal analysis (lead), validation (supporting), writing – original draft (equal), writing – review and editing (supporting). **Berna Akıncı Özyürek:** conceptualization (equal), formal analysis (equal), investigation (equal), project administration (lead), validation (lead), writing – original draft (equal), writing – review and editing (lead). **Metin Dinçer:** conceptualization (equal), formal analysis (equal), investigation (equal), project administration (supporting), supervision (lead), writing – original draft (equal). **Hızır Alı Gümüşler:** data curation (supporting), formal analysis (supporting), investigation (supporting), methodology (supporting), software (lead).

## Conflicts of Interest

The authors declare no conflicts of interest.

## Data Availability

The data for this study is available upon reasonable request from the authors in both original forms derived from the reimbursement investigation and the form that was later combined with the hospital patient system data.

## References

[1] F. Bray , M. Laversanne , H. Sung , et al., “Global Cancer Statistics 2022: GLOBOCAN Estimates of Incidence and Mortality Worldwide for 36 Cancers in 185 Countries,” CA: A Cancer Journal for Clinicians 74 (2024): 229–263, 10.3322/caac.21834. 38572751

[2] A. K. Cangır , P. F. Yumuk , S. D. Sak , et al., “Lung Cancer in Turkey,” Journal of Thoracic Oncology 17 (2022): 1158–1170, 10.1016/j.jtho.2022.06.001. 36192076

[3] Turkish Statistical Institute , “Death and Causes of Death Statistics, 2022.” (2023).

[4] L. A. Torre , R. L. Siegel , E. M. Ward , and A. Jemal , “Global Cancer Incidence and Mortality Rates and Trends—An Update,” Cancer Epidemiology, Biomarkers & Prevention 25 (2016): 16–27, 10.1158/1055-9965.EPI-15-0578. 26667886

[5] G. Luo , Y. Zhang , J. Etxeberria , et al., “Projections of Lung Cancer Incidence by 2035 in 40 Countries Worldwide: Population‐Based Study,” JMIR Public Health and Surveillance 9 (2023): e43651, 10.2196/43651. 36800235 PMC9984998

[6] R. L. Siegel , A. N. Giaquinto , and A. Jemal , “Cancer Statistics, 2024,” CA: A Cancer Journal for Clinicians 74 (2024): 12–49, 10.3322/caac.21820. 38230766

[7] F. Islami , K. D. Miller , R. L. Siegel , et al., “National and State Estimates of Lost Earnings From Cancer Deaths in the United States,” JAMA Oncology 5 (2019): e191460, 10.1001/jamaoncol.2019.1460. 31268465 PMC6613311

[8] Central Bank of The Republic of Turkey , “Exchange Rates.” (n.d.), https://wwwtcmbgovtr/wps/wcm/connect/en/tcmb+en/main+menu/statistics/exchange+rates/indicative+exchange+rates.

[9] X. Zhang , S. Liu , Y. Liu , et al., “Economic Burden for Lung Cancer Survivors in Urban China,” International Journal of Environmental Research and Public Health 14 (2017): 308, 10.3390/ijerph14030308. 28294998 PMC5369144

[10] J. Lortet‐Tieulent , I. Soerjomataram , J. Ferlay , M. Rutherford , E. Weiderpass , and F. Bray , “International Trends in Lung Cancer Incidence by Histological Subtype: Adenocarcinoma Stabilizing in Men but Still Increasing in Women,” Lung Cancer 84 (2014): 13–22, 10.1016/j.lungcan.2014.01.009. 24524818

[11] Y. Zhang , S. Vaccarella , E. Morgan , et al., “Global Variations in Lung Cancer Incidence by Histological Subtype in 2020: A Population‐Based Study,” Lancet Oncology 24 (2023): 1206–1218, 10.1016/S1470-2045(23)00444-8. 37837979

[12] P. C. Tang , M. K. Chan , J. Y. Chung , et al., “Hematopoietic Transcription Factor RUNX1 Is Essential for Promoting Macrophage–Myofibroblast Transition in Non‐Small‐Cell Lung Carcinoma,” Advanced Science 11 (2024), 10.1002/advs.202302203. PMC10767400 37967345

[13] S. Huang , J. Y.‐F. Chung , C. Li , et al., “Cellular Dynamics of Tumor Microenvironment Driving Immunotherapy Resistance in Non‐Small‐Cell Lung Carcinoma,” Cancer Letters 604 (2024): 217272, 10.1016/j.canlet.2024.217272. 39326553

[14] S. Y. Kim , J.‐H. Park , K. H. Kang , et al., “The Economic Burden of Cancer in Korea in 2009,” Asian Pacific Journal of Cancer Prevention 16 (2015): 1295–1301, 10.7314/APJCP.2015.16.3.1295. 25735370

[15] S. J. Seung , M. Hurry , S. Hassan , R. N. Walton , and W. K. Evans , “Cost‐Of‐Illness Study for Non‐Small‐Cell Lung Cancer Using Real‐World Data,” Current Oncology 26 (2019): 102–107, 10.3747/co.26.4555. 31043811 PMC6476449

[16] D. Isla , N. González‐Rojas , D. Nieves , M. Brosa , and H. W. Finnern , “Treatment Patterns, Use of Resources, and Costs of Advanced Non‐Small‐Cell Lung Cancer Patients in Spain: Results From a Delphi Panel,” Clinical and Translational Oncology 13 (2011): 460–471, 10.1007/s12094-011-0683-0. 21775273

[17] J. Park and K. A. Look , “Health Care Expenditure Burden of Cancer Care in the United States,” Inquiry 56 (2019), 10.1177/0046958019880696. PMC6778988 31583928

[18] T. Hofmarcher , A. Manzano García , N. Wilking , and P. Lindgren , “The Disease Burden and Economic Burden of Cancer in 9 Countries in the Middle East and Africa,” Value in Health Regional Issues 37 (2023): 81–87, 10.1016/j.vhri.2023.05.005. 37364406

[19] M. Jakovljevic , P. O. Fernandes , J. P. Teixeira , N. Rancic , Y. Timofeyev , and V. Reshetnikov , “Underlying Differences in Health Spending Within the World Health Organisation Europe Region—Comparing EU15, EU Post‐2004, CIS, EU Candidate, and CARINFONET Countries,” International Journal of Environmental Research and Public Health 16 (2019): 3043, 10.3390/ijerph16173043. 31443381 PMC6747367

